# Editorial: Predictive and prognostic value of liquid biopsy biomarkers in metastatic cancers: from basic science, across high throughput profiling up to clinical practice

**DOI:** 10.3389/fonc.2024.1375711

**Published:** 2024-03-18

**Authors:** Dorota Kwapisz, Patrycja Pawlikowska, Areti Strati

**Affiliations:** ^1^ Department of Biochemistry and Molecular Biology, Centre of Postgraduate Medical Education, Warsaw, Poland; ^2^ Gustave Roussy, Université Paris-Saclay, “Rare Circulating Cells” Translational Platform, CNRS UMS3655 – INSERM US23 AMMICA, Villejuif, France; ^3^ Analysis of Circulating Tumor Cells Lab, Lab of Analytical Chemistry, Department of Chemistry, National and Kapodistrian University of Athens, Athens, Greece

**Keywords:** liquid biopsy, miRNA, CTC, oncology, lncRNA

We are pleased to introduce this Research Topic entitled “*Predictive and Prognostic Value of Liquid Biopsy Biomarkers in Metastatic Cancers: from Basic Science, across High Throughput Profiling up to Clinical Practice*”. This Research Topic includes papers that explore a variety of topics related to liquid biopsy (LB) in the field of oncology. Over the past few years, the interest in LB in oncology has increased significantly, especially after the approval of LB testing in lung cancer (1). Due to its low invasiveness and ease of multiple sample collection, LB has the potential to revolutionize the diagnosis and treatment of cancer and to be a helpful tool in patient follow-up. Large-scale, diverse and comprehensive analyses of various cancer samples are revealing a growing number of potential biomarkers that can be used in diagnosis or treatment (2–4). This Research Topic provides some examples of potential prognostic and predictive biomarkers that can be detected in LB samples highlighting the important role of this technique in oncology.

Lung adenocarcinoma (LUAD) and lung squamous cell carcinoma (LUSC) are the most common subtypes of lung cancer (5). Several new biomarkers with high discriminatory values between LUAD and LUSC have been reported (6). Pan et al. investigated the prognostic value of methyltransferase-like protein 7A (METTL7A) gene expression in lung adenocarcinoma in a total of four different LUAD datasets. METTL7A is associated with the development and progression of various tumor types and has high diagnostic and prognostic value (7). According to Pan et al. when low. METTL7A gene expression was observed in the immune microenvironment it was associated with a poor prognosis [Pan et al.]. The characterization of the lung cancer microenvironment could provide interesting information on the efficacy of immune checkpoint inhibitors (8). In addition, the downregulation of the METTL7A gene may be due to cancer-specific DNA methylation, which plays an important role in tumor programming (9).

The importance and mechanism of action of another potential biomarker for tumor development and progression, namely the chemokine receptor CXCR3 and its ligands, have been described in the review article by Wang et al. CXCR3 is mainly expressed on the surface of activated T cells, B cells, and natural killer cells and plays an essential role in Treg cell accumulation and immunosuppression in tumors [Wang et al., 10]. The differential expression of CXCR3 in different cancer subtypes makes it a potential target for immunotherapy [Wang et al.]. Recent data show that activation of the CXCR3 signaling pathway could be a predictive biomarker in immunotherapy (11).

Long non-coding RNAs (lncRNAs) can regulate cell proliferation, apoptosis, migration, invasion and stem cell maintenance during cancer development (12). The importance of lncRNAs in tumor progression and in particular the role of the lncRNA epidermal growth factor receptor antisense RNA 1 (EGFR-AS1) as a potential biomarker in cancer treatment have been discussed in detail in the article by Zhu et al. The long non-coding RNA EGFR-AS1 mediates epidermal growth factor receptor addiction and modulates treatment response in squamous cell carcinoma, while in renal cancer it enhances the malignant phenotype of RCC cells (13, 14). The clinical application of EGFR-AS1 in human cancers holds considerable potential for cancer diagnosis, prognosis evaluation, and treatment response. However, further studies are needed to clarify the detailed mechanisms of EGFR-AS1 in cancer progression and to validate its usefulness in clinical practice [Zhu et al.].

Metabolic remodeling is a one of a hallmark of cancer and divergen metabolism in tumors has been exploited for diagnostic and therapeutic purposes. The importance of mitochondrial one-carbon metabolism and in particular the increased mRNA expression of its key player methylenetetrahydrofolate dehydrogenase 2 (MTHFD2) has already been highlighted in a meta-analysis of 19 cancer types by Nilsson et al. (15). It appears that MTHFD2 expression is required for cancer cell proliferation since MTHFD2 silencing significantly reduced the proliferation of several cancer cell lines (15). Zhang et al. proposed the involvement of MTHFD2 in the regulation of ferroptosis as a mechanism of tumor adaptation. Specific metabolic changes associated with this adaptation, namely increased MTHFD2 expression, have been proposed by Zhang et al. as a potential prognostic biomarker in triple-negative breast cancer. Previously, MTHFD2 was recognized as one of the potential cancer drivers in breast cancer (16). MTHFD2 expression was shown to be induced in response to TGF-β stimulation in breast cancer cells, suggesting its role in epithelial-to-mesenchymal transition and cancer cell invasion (17). Indeed, similar to the present article by Zhang et al., high expression of MTHFD2 has been shown to be associated with poor clinical prognosis in breast and more recently ovarian cancer (17, 18). Further investigations would open up the utility of this biomarker in clinical practice.

The spectrum of circulating extracellular microRNAs (miRNAs) can be affected by various pathological conditions including cancer, thus opening up their utility as prognostic biomarkers. Indeed, circulating extracellular miRNAs have many features of good biomarkers and can be used to distinguish some specific subtypes of cancers (19). However, specific miRNA signatures are related to the clinical and therapeutic characteristics of the tumors (20). Similarly, diet, regular exercise, or obesity may affect circulating miRNA profiles (21). In the present issue, Niedra et al. identified somatostatin analogues (SSA) – mediated circulating plasma miRNA species associated with growth hormone-secreting pituitary neuroendocrine tumors. The value of this study is increased by the fact that pituitary cancer studies in the context of miRNA are relatively rare compared to other tumor types.

It is known that inflammation can influence cancer development and progression (22). This Research Topic features the work of Wu et al., who evaluated the overall survival (OS) of patients with primary oral squamous cell carcinoma (OSCC) using inflammatory indicators. The authors paid particular attention to the importance of the inflammatory biomarker indicator - lymphocyte to monocyte ratio (LMR) in patients with primary OSCC and its impact on OS. This study confirmed the important role of an inflammatory process in cancer patients. The importance of the LMR ratio has also been assessed in other cancers. In one meta-analysis, pretreatment LMR ratio was found to be a potential prognostic marker for poor prognosis in ovarian cancer patients, while in a second meta-analysis it was suggested that a high LMR ratio may be a useful prognostic marker in colorectal cancer (23, 24).

Multiple and often repeated tissue biopsies are not feasible in clinical practice, therefore, LB represents a non-invasive and easily accessible alternative to assess the tumor characteristics, and constitutes a source of biomarkers during treatment and follow-up. In the LB sample, a rare population of circulating tumor cells (CTCs) may contain clones of tumor cells with high relevance for metastatic progression. Accurate identification and isolation of CTCs remain extremely challenging. Yeo et al. described the detection and isolation of CTCs from peripheral blood samples using an enrichment-free multiparametric high-resolution imaging method.

In conclusion, this Research Topic brings together research findings in the field of LB in oncology, providing an overview of the progress and challenges in developing the utility of LB biomarkers as predictive and prognostic factors.

## Author contributions

DK: Writing – review & editing, Writing – original draft. PP: Writing – review & editing, Writing – original draft. AS: Writing – review & editing, Writing – original draft.
